# Exploring the Interplay of Antimicrobial Properties and Cellular Response in Physically Crosslinked Hyaluronic Acid/ε-Polylysine Hydrogels

**DOI:** 10.3390/polym15081915

**Published:** 2023-04-17

**Authors:** Kristine Aunina, Anna Ramata-Stunda, Ilijana Kovrlija, Eliza Tracuma, Remo Merijs-Meri, Vizma Nikolajeva, Dagnija Loca

**Affiliations:** 1 Rudolfs Cimdins Riga Biomaterials Innovations and Development Centre of RTU, Institute of General Chemical Engineering, Faculty of Materials Science and Applied Chemistry, Riga Technical University, LV-1007 Riga, Latvia; 2 Baltic Biomaterials Centre of Excellence, Headquarters at Riga Technical University, LV-1007 Riga, Latvia; 3 Department of Microbiology and Biotechnology, Faculty of Biology, University of Latvia, LV-1050 Riga, Latvia; 4 Institute of Polymer Materials, Faculty of Materials Science and Applied Chemistry, Riga Technical University, LV-1048 Riga, Latvia

**Keywords:** hyaluronic acid, polylysine, antimicrobial properties, injectable hydrogels, self-healing

## Abstract

The reduction of tissue cytotoxicity and the improvement of cell viability are of utmost significance, particularly in the realm of green chemistry. Despite substantial progress, the threat of local infections remains a concern. Therefore, hydrogel systems that provide mechanical support and a harmonious balance between antimicrobial efficacy and cell viability are greatly needed. Our study explores the preparation of physically crosslinked, injectable, and antimicrobial hydrogels using biocompatible hyaluronic acid (HA) and antimicrobial ε-polylysine (ε-PL) in different weight ratios (10 wt% to 90 wt%). The crosslinking was achieved by forming a polyelectrolyte complex between HA and ε-PL. The influence of HA content on the resulting HA/ε-PL hydrogel physicochemical, mechanical, morphological, rheological, and antimicrobial properties was evaluated, followed by an inspection of their in vitro cytotoxicity and hemocompatibility. Within the study, injectable, self-healing HA/ε-PL hydrogels were developed. All hydrogels showed antimicrobial properties against *S. aureus*, *P. aeruginosa*, *E. coli*, and *C. albicans*, where HA/ε-PL 30:70 (wt%) composition reached nearly 100% killing efficiency. The antimicrobial activity was directly proportional to ε-PL content in the HA/ε-PL hydrogels. A decrease in ε-PL content led to a reduction of antimicrobial efficacy against *S. aureus* and *C. albicans*. Conversely, this decrease in ε-PL content in HA/ε-PL hydrogels was favourable for Balb/c 3T3 cells, leading to the cell viability of 152.57% for HA/ε-PL 70:30 and 142.67% for HA/ε-PL 80:20. The obtained results provide essential insights into the composition of the appropriate hydrogel systems able to provide not only mechanical support but also the antibacterial effect, which can offer opportunities for developing new, patient-safe, and environmentally friendly biomaterials.

## 1. Introduction

Regenerative medicine is an interdisciplinary field that aims to repair damaged tissues. Although many efforts have been devoted to new biomaterial development, most still require open surgeries for their application. Injectable biomaterials (for example, hydrogels) can replace traditional clinical practices while being applied by minimally invasive procedures [1,2]. To be injectable, the material must meet several essential requirements; primarily, it must be biocompatible. In other words, it must not cause negative cell response [1,2,3,4]. Furthermore, it should have appropriate mechanical properties, depending on the site of application of the material in the body [1,5], as well as the high capacity for binding biological fluids, while its degradation products must not cause inflammatory processes [1,4]. For the development of injectable hydrogels, it is possible to use different polymers such as polysaccharides (hyaluronic acid [6,7,8,9], chitosan [10,11,12], alginate [13,14]), polyaminoacids (ε-polylysine [15,16], polyaspartic acid [17]), as well as proteins (silk fibroin [18,19]). The advantages of using them are related not only to the approach of their implantation into the defect (deep and hidden anatomical sites can be filled with the material, thus irregular shape defects can be repaired) but also to the fact that they strongly influence the patient’s recovery time (more minor scars, reduced risk of infection) and physiological level of discomfort during the post-operative period.

Hyaluronic acid (HA) is a natural polysaccharide that is a major component of the extracellular matrix and plays a crucial role in maintaining tissue viscoelasticity [20]. Viscous solutions, with a high molecular weight of HA (>500 kDa), have been used in an array of different wound treatments and for various tissues (e.g., treatment of skin burns). However, due to the undesirable material loss stemming from the minimal control over parameters (swelling, degradation, and mechanics), extensive use of HA solutions is limited in tissue engineering applications. To overcome these disadvantages, significant focus has been placed on developing HA hydrogels for surgical implantation using minimally invasive methods (MIM) [21]. Numerous chemical and physical crosslinking mechanisms have been studied to manufacture injectable HA hydrogels. Recently, Shangzhi Li et al. modified HA with dialdehyde and crosslinked it with cystamine dihydrochloride via the Schiff base crosslinking mechanism. Synthesized hydrogels could be used as drug carriers and scaffolds for bone tissue regeneration [22]. Another strategy for preparing HA hydrogels is to use different crosslinkers such as 1-ethyl-3-[3-dimethylaminopropyl] carbodiimide hydrochloride/N-Hydroxysuccinimide (EDC/NHS) [6,23], glutaraldehyde and 1,4-butanediol diglycidyl ether (BDDE) [24]. These crosslinking agents allow the forming of different covalent bonds (i.e., ether or amide bond) by crosslinking the two polymer chains, with the end goal of creating a hydrogel prompting in better stability in the physiological environment, more controllable biodegradation dynamics than the physically crosslinked hydrogels, and better mechanical properties. Chemically crosslinked hydrogels are also formed by Michael type-addition, Schiff base formation, Diels-Alder “click” reaction, enzyme-induced crosslink, photo-polymerization, etc. On the other hand, crosslinking mechanism of physically crosslinked hydrogels is based on the intramolecular forces capable of forming non-covalent crosslinks, namely electrostatic/ionic interactions, hydrogen bonds, hydrophobic interactions, π-π interactions, etc. The key advantage of the physically crosslinked hydrogels is their biomedical safety, simplicity of fabrication, and prevailing biocompatibility. However, they present low mechanical properties and limited controllability of biodegradation [25].

ε-Polylysine (ε-PL) is a natural polyaminoacid biopolymer synthesized from the *Streptomyces albulus*. Because of its antibacterial nature, in recent years, ε-PL has become more interesting for scientists in biomedicine. Moreover, it is non-toxic [26,27], water-soluble [26,27], biocompatible [27], and stable in high temperatures [26,27], which is an essential aspect for choosing the sterilization method. Antimicrobial properties of ε-PL have been explained as electrostatic interactions between positively charged amino (-NH_3_^+^) groups in polylysine structure and microorganism cell surface [27,28], thus causing damage to the microbial cell membrane [29]. The main factor of polylysine antibacterial activity is the count of protonated NH_3_^+^ groups or the number of L-lysine residues in polylysine structure [30]. D. Alkekhia and A. Shukla [30] showed the molecular weight’s influence on polylysine’s antibacterial properties. By increasing the molecular weight, polylysine’s antibacterial properties increased, and the minimum inhibitory concentration of *S.aureus* decreased [30]. Presently, ε-PL and its derivatives are being used in cancer therapy due to their high stability and drug encapsulation efficiency [31], mainly to make tissue adhesives [32] and bacteria-infected wound treatments [33,34].

As mentioned, hyaluronic acid in the MIM can ensure high water binding capacity while providing the material with the needed mechanical strength [35]. However, further addition of ε-polylysine could provide a highly desired local antibacterial effect [28]. Therefore, HA/ε-PL composites could be used as carriers for local drug delivery and as matrices for bone and cartilage regeneration. Due to the importance of the overall principles of green chemistry (i.e., less hazardous chemical synthesis and designing safer chemicals) producing solvents and auxiliaries is of paramount importance in tissue engineering. [36,37]. Recent studies have shown the high biocompatibility of the biomaterials made of both ε-PL and HA [20,33]. For example, the combination of HA and ε-PL into polyelectrolyte multilayer film has been demonstrated to be a promising approach for antibacterial coatings and local drug delivery [36,38]. Linkages between one carboxylic acid group per disaccharide unit of HA, and one amino group per monomer repeat unit of ε-PL, are being formed via electrostatic interactions, and nano-, micro-, or macro hydrogels can be obtained [37,39]. Physically crosslinked HA/ε-PL nanohydrogels also have potential in drug delivery. Recently, Amato et al. [37] designed physically crosslinked nanogels made of HA/ε-PL and berberine for wound treatments. With the simple mixing technology of HA and ε-PL solutions, it is possible to form drug-loaded nanocarriers with 200 nm dimensions and fast drug release capability (50% within the first 45 min, followed by the sustained release over 24 h). Lawanprasert et al. [40] investigated the encapsulation of small drug molecules and proteins in the HA/ε-PL nanogels for local drug delivery. These novel nanogels have demonstrated high biocompatibility with low systemic toxicity risk. Furthermore, Liu et al. [20] prepared dual-crosslinked hydrogels formed via Schiff base and enzymatic catalytic reaction, using oxidized HA and ε-PL, for the wound treatments. Histological studies indicated that advanced hydrogels are antibacterial—they effectively kill bacteria in the wound and promote wound healing (high cell viability >90% on the third day) [20]. Salma-Ancane et al. [41] compared three different HA and ε-PL ratios (HA to ε-PL mass ratio of 40:60 wt%, 50:50 wt%, 60:40 wt%) of physically and chemically cross-linked hydrogels to show the link between the different cross-linking mechanisms and the hydrogel chemical, physical, and biological properties. In this study, physically crosslinked hydrogels confirmed an excellent antimicrobial effect against the gram-negative bacteria *Escherichia coli* (*E. coli*), while cell-viability studies indicated pronounced cytotoxic effects of all three physically crosslinked hydrogel compositions [41].

In the current research, the main goal was to prepare physically crosslinked hydrogels with a decreased amount of ε-PL and to answer the most important question: does the newly formed range of compositions still provide the antimicrobial effect while preserving favourable conditions for in vitro cell viability? Using the novel green chemistry approach, HA/ε-PL hydrogels have been physically crosslinked with ε-PL content of 30 wt%, 20 wt% and 10 wt% and subsequently analyzed. The use of safe methods and materials complying the aforementioned principles of green chemistry approach, to eliminate the high risk of tissue cytotoxicity and increase cell viability were one of the priorities. Moreover, we have gone one step further from the current literature by analysing an abundance of different ratios of HA and ε-PL (HA to ε-PL mass ratio from 10:90 wt% to 90:10 wt%) and by evaluating the antimicrobial activity of all the compositions against *E. coli*, *S. aureus*, *P. aeruginosa*, and *C. albicans.* Furthermore, the influence of ɛ-PL content in hydrogels on their in vitro biocompatibility with Balb/c 3T3 cells was evaluated. Within the study, we have successfully shown the indispensable information of the effect of HA content and its interaction with ε-PL, on the set of presented properties. The results authenticated specifically designed hydrogels that are biocompatible, antimicrobial and have good mechanical properties while being synthesised without chemical cross-linking agents, special conditions or modifications. Moreover, a set of these new hydrogels have a great potential in minimally invasive surgical approach that could minimize local infection risks.

## 2. Materials and Methods

### 2.1. Materials

Contipro a.s. (Dolní Dobrouč, Czech Republic) supplied the sodium hyaluronate (reffered later as hyaluronic acid or HA, cosmetic grade, >97.3%, molecular weight 1.71 MDa, 600-01-12), Zhengzhou Bainafo Bioengineering Co., Ltd. (Henan, China) provided the ε-polylysine (ε-PL, >95%, molecular weight 3850 g/mol). Tablets for phosphate buffer saline (PBS, pH 7.2–7.6, PCode 1003151781) preparation, Dulbecco’s modified Eagle’s medium (DMEM, Gibco, 1056606), calf serum (Sigma, C8056), penicillin/streptomycin solution (Sigma, P4333), trypsin/ ethylenediaminetetraacetic acid (EDTA, Sigma, T4049), sodium dodecyl sulphate (Sigma, L3771) were purchased from Sigma-Aldrich (St. Louis, MO, USA). Neutral red solution and glacial acetic acid were ordered from Sigma-Aldric (Irvine, UK, N2889 and A6283, respectively). Sodium chloride (NaCl, >99.5%, K51852004209) was ordered from Sigma-Aldrich (Steinheim, Germany). The ethanol solution was purchased from Kalsnavas Elevators, Latvia. Tryptic soy broth (CAS 4021552) and malt extract agar (CAS 70145) were ordered from Biolife (Milan, Italy). Merck (Darmstadt, Germany) supplied malt extract broth (CAS 70146). Sanofi Diagnostics Pasteur (Marnes-la-Coquette, France) provided plate count agar (CAS 64475). Life Technologies Corp. (Eugene, OR, USA) supplied cell viability dyes, SYTO 9 and propidium iodide (S34854 and R37108). Deionised water (DI) was used throughout the study.

### 2.2. Preparation of HA/ε-PL Hydrogels

HA/ε-PL hydrogels, with different HA to ε-PL weight ratios (wt%) (from 90:10 to 10:90), were prepared by physically crosslinking two biopolymers in an aqueous medium. The solid to liquid phase ratio remained constant at 1:2.5 for all compositions. Immediately after preparation, hydrogels were used for injectability and rheological measurements. For all other experiments, hydrogels were formed in the stainless-steel moulds (h = 5 mm, d = 10 mm for assessment of biological properties and h = 10 mm, d = 10 mm for characterisation of chemical and physical properties) and left to crosslink for one hour at room temperature.

### 2.3. Sterilization of HA/ε-PL Hydrogels

Sterilisation was performed in an autoclave (ELARA11, Netherlands) at 105 °C for 4 min under 179.5 kPa. Sterilised samples were kept at 4 °C until further characterisation.

### 2.4. Lyophilization of HA/ε-PL Hydrogels

Samples were frozen at −26 °C and lyophilised using Martin Christ laboratory lyophilisation (BETA 2-8 LCSplus, Germany) at −85 °C for 72 h. Lyophilised samples were used for gel fraction, swelling behaviour, Fourier transform infrared spectroscopy (FT-IR), Scanning electron microscopy (SEM) and X-ray microtomography (μ-CT) analysis.

### 2.5. Physicochemical Characterisation

#### 2.5.1. Swelling Behaviour

The swelling behaviour of lyophilised HA/ɛ-PL composites was evaluated in 20 mL of deionised water (DI) at 37 °C and 100 rpm. Freeze-dried samples were weighed and soaked in the DI. The swollen hydrogels were periodically taken out of DI and weighed after removing the excess water. Measurements were performed every hour for the first four hours and then every week for four weeks. The swelling ratio (SR) of hydrogels at different time intervals was calculated according to the following equation:

$$SR=\frac{{(w}_{s}-w_{d})}{w_{d}}\times 100\%,$$

where $w_{d}$ is the weight of the freeze-dried hydrogel sample, and $w_{s}$ is the weight of the swollen hydrogel sample. The results are shown as an average value ± standard deviation from three replicates of each prepared hydrogel composition.

#### 2.5.2. Gel Fraction

The gel fraction (GF) of lyophilised samples was determined by measuring their insoluble part. Samples were immersed in 200 mL of deionised water (DI) at 37 °C and 100 rpm for 48 h. Afterwards, samples were removed from DI, lyophilised, and weighed again. The gel fraction was calculated as follows:

$$GF=\frac{w_{d}}{w_{i}}\times 100\%,$$

where $w_{d}$ is the weight of the freeze-dried hydrogel sample and $w_{i}$ is the weight of freeze-dried sample after swelling in DI water. The results are shown as an average value ± standard deviation from three replicates of each prepared hydrogel composition.

#### 2.5.3. Injection Force

The injectability of prepared hydrogels was determined using self-designed injectability equipment (Supplementary Material Figure S1). Tinius Olsen 25 ST (Horsham, PA, USA), a multifunctional, mechanical materials testing machine was used in a compression mode with a load cell of 5 kN. 1.5 g of hydrogel was placed in a 5 mL Luer-lock syringe. Injection force (IF) was measured at a rate of 1 mm/s through the syringe needle of 14 G (1.6 mm inner diameter and 70 mm in length). The results were shown as an average value ± standard deviation from 5 replicates.

#### 2.5.4. Molecular Structure

The functional groups of HA, ε-PL and lyophilised HA/ε-PL hydrogel samples were identified by Fourier transform infrared spectroscopy (FT-IR). FT-IR spectra were obtained using the Varian 800 FT-IR spectrometer. Before measurements, HA/ε-PL lyophilized composites were ground to a powder composition. Three mg of sample and 300 mg of spectroscopic grade KBr were mixed using a ball mill (Mini-Mill PULVERISETTE 23 (FRITCH, Idar-Oberstein, Germany)). KBr and hydrogel pellets with a diameter of 13 mm were prepared using an isostatic press (P/O/Weber Laborpresstechnik, Remshalden, Germany) and analysed. FT-IR spectra were recorded at 30 scans/min, resolution of 4 cm^−1^ and a wavenumber range from 4000 cm^−1^ to 400 cm^−1^. Before each measurement, the background air spectrum was obtained. The acquired spectrum was normalized using Origin 2018. Normalisation was optimised with a default setting of the software algorithm (range from 0 to 1 corresponding to minimum and maximum values of raw absorbance intensities, respectively). Normalised data were used to determine the ratio of NH_3_^+^/NH_2_, indicatively evaluating the overall charge status of prepared HA/ε-PL composites [41].

#### 2.5.5. Morphology

Tescan Mira\LMU (Czech Republic) scanning electron microscope was used to examine the surface morphology and the inner structure of prepared HA/ε-PL hydrogels at an acceleration voltage of 10 kV. The hydrogel samples were fixed on aluminium pin stubs by using double-sided adhesive carbon tape. Each sample was sputter coated with a 15 nm thin gold layer before imaging using Emitech K550X (Quorum Technologies, Ashford, Kent, UK) sputter coater. The size of pores was computed manually from 25 pores per sample on several randomly chosen collected SEM images. The shape of the pores was approximated with ellipses by using two radii (Y as the longest and representing the meridian; X as the shortest and representing the equatorial axis) being determined for each pore using the imageJ© program.

Scanco Medical μCT50 (Bassersdorf, Switzerland), micro-computer tomography was used to examine the surface morphology and the inner structure of prepared HA/ε-PL hydrogels. Computed tomography images were acquired at 70 kVp and 114 μA. The aluminium filter of 0.5 mm was used for all hydrogel compositions. Hydrogel’s porosity in the sample was calculated based on the scanned cross-sections at the lower, middle and upper area by using the ratio of the object to the total volume of the sample. The results were shown as an average value ± standard deviation from 3 replicates.

#### 2.5.6. Oscillatory Rheology

Rheological properties of HA/ε-PL hydrogels were determined in oscillation mode on the HR20 Discovery Hybrid rheometer from TA Instruments (New Castle, USA). Rheometer was equipped with a parallel plate geometry of 20 mm. 1 mm of measuring gap was used for all HA/ε-PL ratios. Samples were prepared as described above (see Section 2.2) and immediately placed on the bottom plate with a temperature of 25 °C. A layer of silicone oil was applied to the edges of the sample to reduce evaporation. Before each measurement, hydrogel samples were equilibrated for 120 s. A frequency sweep test was carried out by subjecting the sample to 1% strain within the frequency range from 1 to 100 Hz. The storage modulus at 1 Hz was chosen to determine the stiffness of prepared hydrogel samples. An amplitude sweep test was conducted by exposing the hydrogels to a frequency of 1 Hz within the strain range of 0.01% to 100%. A time sweep test was used to determine the point at which hydrogels crosslink. The appropriate parameters from the frequency (linear viscoelastic region) of this measurement were selected from frequency (1 Hz) and amplitude sweep (1%) tests. Time sweeps were performed by subjecting the sample to the frequency of 1 Hz and strain of 1% for 300 s. The self-healing potential of prepared HA/ε-PL hydrogels was determined by cutting the sample in two pieces and leaving it to crosslink for one hour at room temperature. After one hour, the time sweep test was performed, and the obtained results were compared with those previously measured for non-cut samples. To visualise the self-healing process, one hydrogel sample was coloured with food colour, and one was uncoloured. Both samples were cut into two pieces and placed together. The self-healing process was observed at different time points (20 min, 1 h, 3 h, 14 h). The recovery properties of the prepared hydrogel samples were determined by changing the strain from 1% to 100% for three cycles at a constant frequency of 1 Hz. All experiments were performed in triplicate, and the results were presented as an average ± standard deviation.

### 2.6. In Vitro Antimicrobial Characterisation

Antimicrobial properties of prepared HA/ε-PL hydrogels against diverse microorganisms were investigated by the antimicrobial activity evaluation in microbial suspension and zone-of-inhibition test according to a modified ASTM E2149-10 method (“Standard Test Method for Determining the Antimicrobial Activity of Immobilized Antimicrobial Agents Under Dynamic Contact Conditions”). In this study, we used gram-positive bacteria *Staphylococcus aureus* (*S. aureus*), gram-negative bacteria *Pseudomonas aeruginosa* (*P. aeruginosa*) and *Escherichia coli* (*E. coli*) and yeast *Candida albicans* (*C. albicans*). Fresh 24 h shaken cultures of *E. coli* MSCL 332 (ATCC 25922), *S. aureus* MSCL 334 (ATCC 6538P) and *P. aeruginosa* MSCL 331 (ATCC 9027), grown in sterile Tryptic soy broth at 36 ± 1 °C, were used in the experiments. Yeast-like fungus *C. albicans* MSCL 378 (ATCC 10261) was grown in sterile Malt extract broth at 36 ± 1 °C for 48 h. Cultures were diluted with sterile water until they reached the absorbance of A_540_ = 0.16 ± 0.02, which corresponded to approximately 10^6^ CFU mL^−1^.

#### 2.6.1. Agar Well Diffusion Method

Plate count agar (Bio-Rad, Marnes-la-Coquette, France) plates for bacteria and Malt extract agar (Merck Millipore, Darmstadt, Germany) plates for fungi were inoculated with a confluent lawn of microorganisms. 8 mm diameter wells were bored in the agar medium, and HA/ε-PL samples (with a diameter of 7 mm) were placed in triplicate in agar wells. Gentamicin (KRKA, Slovenia; 10 mg/mL, 70 μL) was used as a positive control. Bacteria were cultivated on an agar medium at 36 ± 1 °C for 24 h, and *C. albicans* was cultivated at 36 ± 1 °C for 48 h. Sterile zones of inhibition were measured using a millimetre-scale ruler.

#### 2.6.2. Dynamic Contact Test

HA/ε-PL hydrogel samples were placed in a 50 mL tube filled with 5 mL of microbial working suspension in potassium phosphate buffer. A series of dilutions were subsequently prepared from the inoculum tube that did not contain the HA/ε-PL hydrogel sample and inoculated into Petri dishes on agar media. The experiment was carried out in triplicate to confirm the initial microbial concentration in colony-forming units (CFU) mL^−1^. All prepared samples were incubated at 35 ± 2 °C, shaking (200 rpm) for 1 h ± 5 min. Following the same procedure that was carried out for the sample without HA/ε-PL hydrogel, samples with the hydrogels were diluted in triplicate and inoculated into Petri dishes, with Plate count agar for bacteria and Malt extract agar for *C. albicans* to estimate the number of CFU mL^−1^. Prepared Petri dishes were incubated at 35 ± 2 °C for 24 h. Antimicrobial properties were investigated according to a modified ASTM E2149-10 method. The mean CFU mL^−1^ and microbial reduction were calculated in the following manner:

$$Log10\;microbial\;reduction=Log10\left(B\right)-Log10\left(A\right),$$

where *A* = CFU mL^−1^ for the tube containing HA/ε-PL hydrogel samples after 1 h contact time, and *B* = CFU mL^−1^ for not containing HA/ε-PL hydrogel samples after 1 h contact time. The results were shown as an average value ± standard deviation from 3 replicates for each hydrogel composition.

### 2.7. In Vitro Cytotoxicity Assay

BALB/c 3T3 cell line (ATCC) was used for cytotoxicity assays. Cells were grown in Dulbecco’s modified Eagle’s medium (DMEM, Sigma, Saint Louis, MO, USA), supplemented with 10% (*v*/*v*) calf serum (Sigma, Saint Louis, MO, USA) and 100 μg mL^−1^ of streptomycin, and 100 μg mL^−1^ of penicillin, at 37 °C within a humidified 5% CO_2_ atmosphere. Cells were detached and passaged using 0.25% (*w*/*v*) of trypsin/EDTA (Sigma, Saint Louis, MO, USA). For all experiments, cell seeding density was 3 × 10^4^ cells cm^−2^. Throughout the cytotoxicity testing, previously reported data [41] were complemented with additional replicates and compared with new compositions of HA/-PL hydrogels.

#### 2.7.1. Extract Test

An extract test was performed to assess the potentially toxic effects of hydrogel components. BALB/c 3T3 cells were seeded in 96-well plates and incubated for 24 h to allow them to attach and start proliferating. Hydrogel samples were washed with phosphate buffered saline (PBS, pH 7.4) and extracted with cell cultivation media for 24 h at 37 °C. The biomaterial to media ratio used for extraction was 1:5 (0.2 g per 1 mL media), except for HA/ε-PL 80:20 and HA/ε-PL 90:10 ratios where they were 1:20 and 1:50, due to swelling of hyaluronic acid during extraction period. Extracts were then collected and diluted with cultivation media to 12.5%, 25% and 50% (*v*/*v*). 0.5 mL of diluted extract was added to the corresponding cell culture wells of a 24-well plate and incubated for 24 h. Cells incubated without hydrogel samples were used as untreated controls, while sodium dodecyl sulphate was used as the positive (cytotoxic) control. Phase contrast microscopy was used to monitor cell cultures and changes in the cell confluence and morphology. After incubation, cultivation media was removed, and cells were washed with PBS. Neutral red (Sigma, Saint Louis, MO, USA) working solution (25 µg mL^−1^) in 5% serum containing cultivation media was added, and cell cultures were incubated for 3 h at 37 °C and 5% CO_2_. Neutral red media was removed, and 1% glacial acetic acid/50% ethanol solution was added to extract the accumulated dye in the viable cells. After 20 min of incubation at room temperature, absorption at 540 nm was measured. Changes in the cell viability were calculated using the following equation:

$$Cell\;viability\left(\%\right)=\frac{{Abs}_{540nm}\left(treatment)-{Abs}_{540nm}(background\right)}{{Abs}_{540nm}\left(untreated\;control)-{Abs}_{540nm}(background\right)}$$

Five replicate samples of extracts from every physically crosslinked hydrogel composition and four replicates of extracts from each chemically crosslinked hydrogel composition were analysed. The results were represented as an average value ± standard deviation.

#### 2.7.2. Direct Contact Assay

Balb/c 3T3 cells were seeded in 6-well plates and incubated for 24 h. Hydrogel samples (100 mg per well) were washed three times with PBS and preincubated for 1 h in 5 mL of cell cultivation media. Subsequently, hydrogels were transferred to cell cultures and incubated for 24 h. After incubation, media and hydrogel samples were removed, and cells were washed with PBS. A neutral red solution was added, and its uptake was measured as described before (see Section 2.7.1). Cells incubated without samples were used as untreated controls, while sodium dodecyl sulphate was used as the positive (cytotoxic) control. Phase contrast microscopy was used to monitor the cell cultures and changes in cell confluence and morphology. Five replicate samples from each prepared hydrogel composition were analysed, and results were represented as an average value ± standard deviation.

#### 2.7.3. Cell Viability Staining

Balb/c 3T3 cells were seeded on the 4-well chamber slides and incubated for 24 h. Hydrogel samples (30 mg per chamber) were prepared as described before (see Section 2.7.2) and added to the cell cultures. After 24 h of incubation, the media was discarded, and cells were washed with PBS. A mixture of fluorescent cell viability dyes (5 µM SYTO 9 dye and 30 µM propidium iodide) was added to the cells and incubated for 15 min at 37 °C, 5% CO_2_ in the dark. After incubation, cells were washed with PBS and imaged using Leica DMI400B inverted fluorescence microscope.

### 2.8. Hemocompatibility of Hydrogels

A hemolysis test was performed to assess the hemocompatibility of hydrogels in a direct contact test. Blood from healthy donors was collected in Monovette vacutainers containing EDTA. Blood was diluted with 0.9% sodium chloride solution (4:5 ratio by volume). Hydrogel samples were washed three times with PBS (pH 7.4) and added to 15 mL tubes containing fresh 9.8 mL PBS, which were then incubated at 37 °C and 5% CO_2_ for 30 min. In the case of hydrogel extracts, 9.8 mL of 20% extracts were used. Extracts were prepared as described in Section 2.7.1. 0.2 mL of diluted blood was added to each tube and incubated at 37 °C and 5% CO_2_ for 1 h and 6 h. PBS was used as a negative control and deionised water as a positive control. After incubation, tubes were centrifuged at 2000 rpm for 5 min, the supernatants were collected, and the absorbance was measured at a wavelength of 545 nm in a Tecan Infinite Pro 200 multimode plate reader.

The hemolytic ratio (HR) was calculated using the following equation:

$$HR(\%)=\frac{\left(Abs\left(sample\right)-Abs\left(negative\;control\right)\right)}{\left(Abs(positive\;control-Abs\left(negative\;control\right)\right)}\times 100$$

Hemocompatibility studies were performed in accordance with the approval of the Committee of research ethics of the Institute of Cardiology and Regenerative Medicine, University of Latvia, No 3/2021.

### 2.9. Statistical Analysis

Obtained results were presented as an average value ± standard deviation of at least three replicates. Statistical significance between all ratios of prepared composition was determined using an unpaired Student’s t-test with a significant level at 95% (*p* < 0.05). One-way ANOVA with Tukey HSD (Honestly Significant Difference) post-hoc test was used to evaluate the statistical significance of in vitro tests. Significance was found if the *p*-value was less than 0.05 (*p* < 0.05).

## 3. Results and Discussion

### 3.1. Influence of HA/ε-PL Ratio on Hydrogel Swelling Behaviour and Gel Fraction

**Figure 1:**
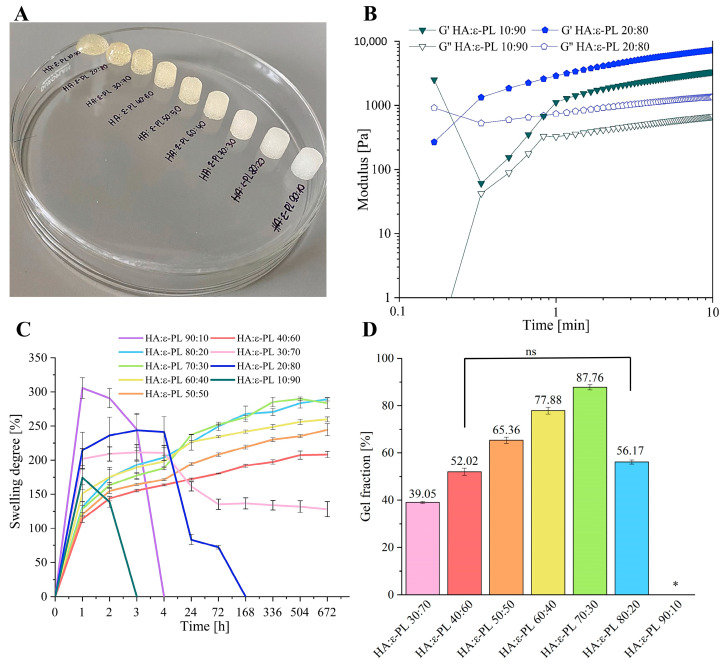
(**A**) Hydrogels containing different content of HA (from 10 wt% up to 90 wt% of HA) (**B**) gelation process of hydrogels containing 10 wt% and 20 wt% of HA at 25 °C (**C**) Swelling behaviour of HA/ε-PL composites (**D**) Average gel fraction values of HA/ε-PL composites. *—samples dissolved, (*n* = 3, ns—no significant difference between the samples).

The most likely binding mechanism of the prepared hydrogels is the electrostatic interaction between the carboxylate group in the HA structure and the amino group in the ε-PL structure, as well as inter-molecular hydrogen bonds in the HA structure [6,41]. Since these are physical linkages and hydrogels are non-covalently crosslinked, the interaction could easily be disturbed by increasing the temperature, changing the pH of a surrounding medium, or mechanically affecting the hydrogels [42]. In order to evaluate the stability of prepared hydrogels their swelling behaviour, as well as the gel fraction were examined. HA/ε-PL hydrogel series with HA content from 10 wt% up to 90 wt% were prepared and compared (Figure 1). By increasing the HA content, it was observed that hydrogels became stiffer and less transparent. Compositions with HA content of 10 wt% and 20 wt%, after one hour of crosslinking, did not retain the cylindrical form and could be easily deformed (Figure 1A). Gel formation occurs also for HA/PL = 10/90 according to Figure 1B. However, for this wt% ratio, either the gel formation point is at a time of less than 0.1–1 min, or the system initially has G’ > G″ due to the macromolecular entanglements of hyaluronic acid (Figure 1B). Most probably, the HA/PL = 10/90 does not keep its shape because it either has no yield stress or has low yield stress. The cross-over point is usually considered as the gel point or hydrogel formation point [43,44]. It was also found that the cross-over point of G’ and G″ curves for hydrogels containing 20 wt% of HA could be observed, indicating that HA/ε-PL hydrogels require at least 20 wt% of HA to form physical crosslinks. Furthermore, swelling experiments showed that hydrogels containing 10 wt% of HA, decomposed in the aqueous medium already after two hours, while hydrogels containing 20 wt% of HA lost their cohesion and completely decomposed after 72 h (Figure 1C). Thus, hydrogels with HA content from 30 wt% up to 90 wt% were selected for further characterization.

Water and different medium absorption is one of the main properties to describe the behaviour of hydrogels in biological conditions. It includes nutrient supply, oxygen transmission, as well as the removal of cellular waste products from the hydrogels [45]. The water absorption kinetics of lyophilized hydrogels were determined for 672 h. Obtained results revealed that even though the samples with HA content of 90 wt% were selected for further tests, they decomposed after three hours, which could be explained by the fact that the hydrogels did not contain enough ε-PL amino groups to form sufficient physical crosslinks that are necessary for stable composite preparation. Hydrogels containing 30 wt% of HA reached the maximum swelling degree of 201.9 ± 15.0% in the first hour and retained it for up to four hours. Between 4 h and 72 h partial dissolution occurred (most probably some part of ε-PL dissolved) and the swelling degree declined. However, after 72 h samples reached again the swelling equilibrium, with the swelling degree equal to 135.2 ± 7.6%. Hydrogels containing 40 wt% of HA slowly swelled and reached the equilibrium in 504 h where the swelling degree was equal to 207.3 ± 5.9%. The equilibrium of swollen hydrogels, with HA content from 50 to 80 wt%, was reached after 168 h, where the swelling degree value for HA/ε-PL 50:50 composite was 219.2 ± 2.9%, for HA/ε-PL 60:40—241.9 ± 2.3%, for HA/ε-PL 70:30—262.6 ± 2.6%, and for HA/ε-PL 80:20—267.9 ± 11.3%. Since the water soaking capacity is over 200% these hydrogels may be described as superabsorbent hydrogels [46]. Hydrogel compositions in which HA content is at least 30 wt% retained water in its structure for more than one month, providing appropriate conditions for acute and chronic wound treatment [47]. The obtained gel fraction results showed that samples with HA content of 70 wt% exhibited the highest value of gel fraction (87.76 ± 1.09%) (Figure 1D), indicating the highest crosslinking degree between HA and ε-PL. It was also observed that if HA content in hydrogels is <40 wt%, already 70% of prepared compositions dissolved after 48 h incubation in DI.

Hence, it can be concluded that the lowest amount of HA needed to form a stable HA/ε-PL hydrogel should be 40 wt%. Moreover, obtained results also revealed that, if HA content in the hydrogels exceeded 80 wt%, physical crosslinks in DI were disrupted within 3 h, indicating that an insufficient amount of ε-PL amino groups were introduced in the composite to form a stable hydrogel.

### 3.2. Influence of the HA/ε-PL Ratio on Hydrogel Crosslinking Ability, Mechanical and Rheological Properties

**Figure 2:**
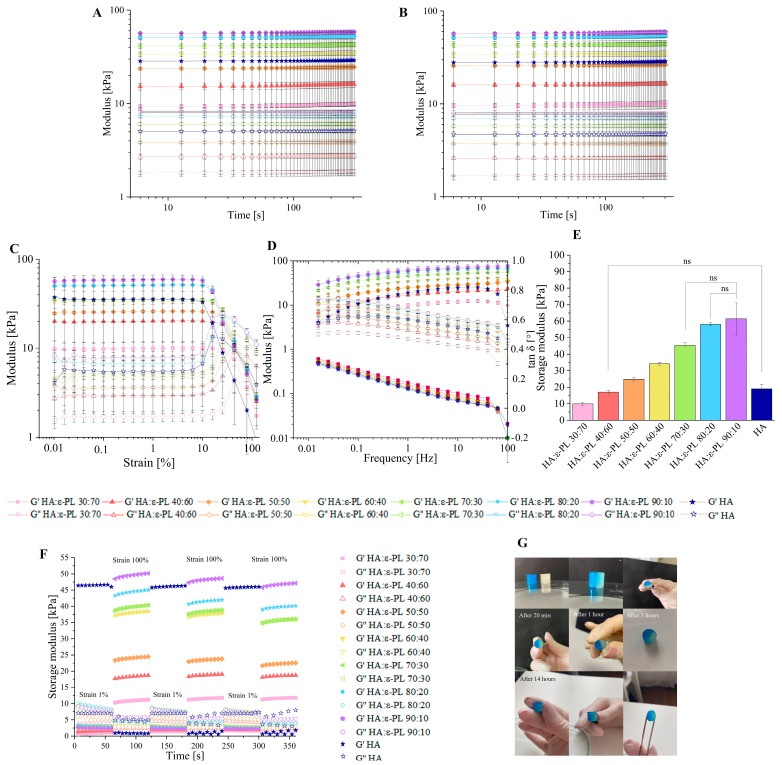
(**A**) Formation process of HA/ε-PL hydrogels; (**B**) Time sweep experiment to determine the self-healing ability of HA/ε-PL hydrogels; (**C**) Amplitude sweep test of HA/ε-PL hydrogels; (**D**) Frequency sweep test of HA/ε-PL hydrogels; (**E**) Stiffness of prepared HA/ε-PL hydrogel samples (*n* = 5; ns = not significant); (**F**) Recovery test of HA/ε-PL hydrogels; (**G**) Visualized process of self-healing. The total wt% of HA/ε-PL hydrogels was 100. HA represents HA/ε-PL 100:00 used as a control.

To track the crosslinking process of HA/ε-PL compositions a time sweep test was performed. The obtained results (Figure 2A) showed that by increasing the HA content in the hydrogel composition, the value of storage modulus (G’) increased, from which it can be concluded that the prepared hydrogels became stiffer, leading to higher mechanical properties [24,48,49]. Since the G’, for compositions where HA ranged from 30 up to 90 wt%, was always greater than the value of the loss modulus (G″), it can be concluded that physical crosslinks were formed immediately after the liquid phase addition to the solid phase. Moreover, the time sweep test allows for estimating the potential self-healing ability. Physically crosslinked hydrogels could be characterized as self-healable or reversible hydrogel systems [42,50]. This aspect is very important for biomaterials due to the constant dynamic environment in the body. In the treatment of the wound, it is necessary that the material recovers itself and regains its function after damage or mechanical disruption [47]. To illustrate the self-healing ability of prepared samples, hydrogels were cut into two pieces, one hydrogel part was coloured in blue, while the other part was left uncoloured. Both parts were placed together (Figure 2G) and left to crosslink. After three hours the cut was less visible, but after 14 h there was no sign of the cut and the hydrogel was completely recovered, indicating that such a hydrogel system could be able to heal and maintain its functions in a dynamic environment of the body. The obtained results of the modulus G’ had the same value as it had before the cut for all HA/ε-PL hydrogels compositions, which proved the high self-healing potential of samples (Figure 2A,B). The self-healing ability of hydrogels could be explained by non-covalent interactions in hydrogel structure. Non-covalent links, in this case, electrostatic interaction and hydrogen bonds, are highly flexible and can be easily destroyed and reconstructed [51]. In order to determine the recovery properties of prepared HA/ε-PL hydrogel samples, the continuous step-strain method was used (Figure 2F). As the strain value became equal to 100%, G’ rapidly decreased approaching the value of G″, indicating that the hydrogel structure was destroyed. When the strain value was decreased to 1%, the value of G’ returned to the original value. It was found that hydrogel samples could recover their own structure for at least three cycles, indicating self-healing capability. To determine the mechanical properties of prepared HA/ε-PL hydrogel samples, a strain sweep test was executed (Figure 2D). All hydrogel series showed an obvious linear viscoelastic region (LVR) up to the ε ≈ 10% strain, which is shown as a plateau region in the graph. Surprisingly, by increasing the HA content in the HA/ε-PL hydrogels, the G’ and G″ cross-over point, has decreased from 69.8% in the case of HA/ε-PL 30:70 down to 22.4% in the case of HA/ε-PL 90:10, indicating reduced durability to gel-liquid transition. Moreover, after reaching the cross-over point hydrogels started to behave as fluid-like substances, indicating that the crosslinked network was disrupted. Furthermore, the cross-over point modulus has increased by increasing the HA content in the hydrogel composition, revealing that the hyaluronic acid content has a dominant impact on the mechanical properties and stability of prepared HA/ε-PL hydrogel systems. By increasing the hyaluronic acid content in the prepared samples, the hydrogels were less stable in higher deformation values, but the mechanical properties were higher, as shown by the module values.

To describe the hydrogel behaviour after their injection into the living tissues, a frequency sweep test was performed (Figure 2D). Frequency sweep test was carried out at 1 Hz, based on the amplitude sweep experiments. Obtained results showed that G’ values were always greater than G″ values, indicating that all tested hydrogels have solid-like structure. Furthermore, obtained tan δ values for all HA/ε-PL hydrogel series were always less than 1, at all frequencies (tan δ < 1), indicating the dominance of elastic properties [52]. Moreover, G’ and G″ curve cross-point was not observed, indicating that all tested ratios of HA/ε-PL hydrogels have a high cross-linking degree at all frequency values. By increasing HA content in the hydrogels, G’ values increased, indicating a less viscous flow behaviour [53]. A frequency sweep test was also used to determine the stiffness of prepared HA/ε-PL hydrogels (Figure 2E). It was found that by increasing HA content in the HA/ε-PL hydrogels, storage modulus values increased from 9.93 ± 0.89 kPa in case of HA:ε-PL 30:70, up to 61.4 ± 9.72 kPa in case of HA:ε-PL 90:10, showing stiffer hydrogel structure and thus higher mechanical properties. Moreover, no significant (*p* > 0.05) differences were found between the stiffness results for hydrogels with HA content of 70, 80 and 90 wt%. These findings go well together with observed values in gel fraction experiments in which the highest crosslinking degree was detected for samples with HA content of 70 wt%. Finally, the range of stiffness values clearly showed that the developed hydrogels have potential applications in tissue engineering, as the modulus values found are similar to those of biological tissues [54]. Throughout the tests pure HA diluted in DI water in ratio 1:2.5 *w*/*v* was used as a control.

### 3.3. Injection Force Measurements

**Figure 3:**
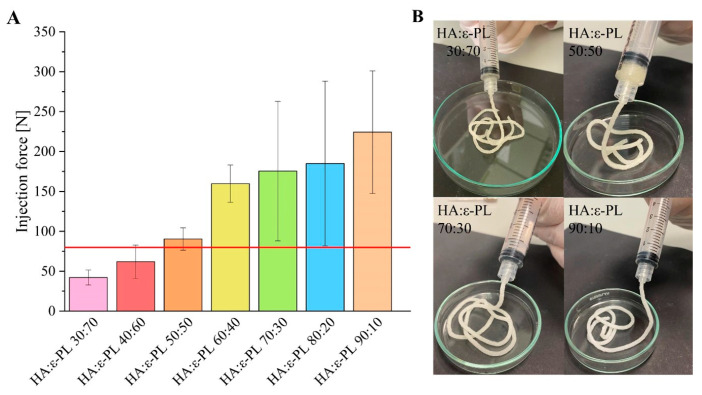
(**A**) Average injection force values of HA/ε-PL hydrogels and the maximum injection force value (red line). (**B**) Hydrogel injection through a syringe.

An injection force (IF) of 79.8 N is considered the maximum force that can be generated by both genders (shown as a red horizontal line in Figure 3A) [55]. As there is no standard method for determining injectability, it is possible to reduce the friction and the final IF value by varying the inner diameter and length of the needle (increased length and diameter lead to higher IF) [55,56,57]. On the other hand, reduced inner diameter of the syringe would increase the pressure at the same force and even more reduce the effective viscosity of the gel. Furthermore, the injection force value is also dependent on the viscosity [56] and density [55] of the material, as well as on the injection rate [56]. Obtained results revealed that by increasing the HA content in the prepared HA/ε-PL hydrogels the value of injection force increased from 42.1 ± 9.28 N in the case of HA/ε-PL 30:70 to 224 ± 76.8 in the case of HA/ε-PL 90:10 (Figure 3A,B). These results once again confirmed the effects found when determining the gel fraction and swelling, indicating that the increase of HA content in hydrogels facilitates the formation of a higher crosslinked polymer network.

### 3.4. Chemical Characterization

**Figure 4:**
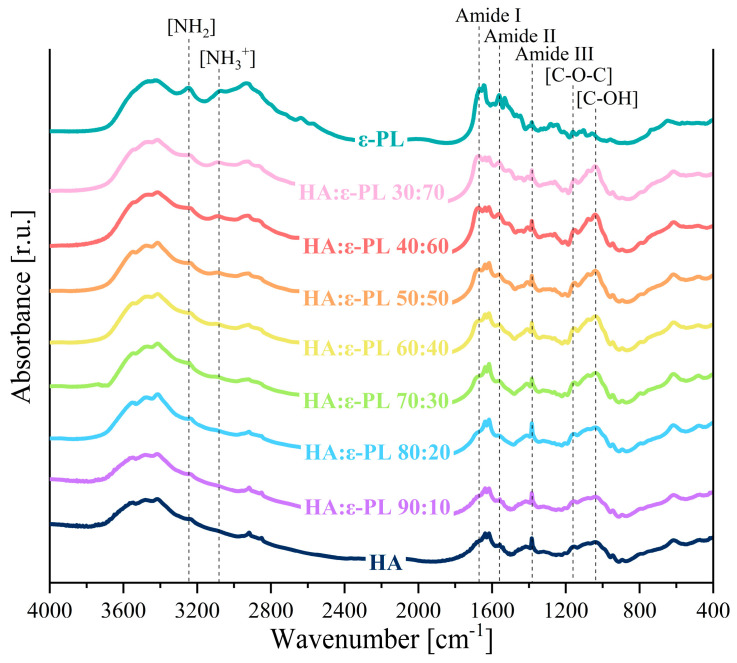
FT-IR spectrum of HA/ε-PL composites.

FT-IR spectroscopy analysis was performed in order to further identify the possible cross-linking mechanisms of HA and ε-PL. In the acquired FT-IR spectrum (Figure 4) the absorption bands between 3246 cm^−1^ and 3081 cm^−1^ that correspond to NH_2_ stretching vibrations and protonated NH_3_^+^, in the ε-PL structure were clearly discerned [58]. At the same time, the HA bands characteristic of amide II and amide III were observed at 1558 cm^−1^ and 1389 cm^−1^ [59,60,61,62], while the bands at 1161 cm^−1^, 1081 cm^−1^, and 1037 cm^−1^ corresponding to the C–O–C, C–O, and C–OH groups, respectively, were also present [60,61]. Additional absorbance bands at 1669 cm^−1^, 1631 cm^−1^, and 1384 cm^−1^ can be assigned to (C=O) and (C–O) stretching vibrations of carboxyl groups of polylysine and hyaluronic acid [59,60]. However, the lack of absorbance band corresponding to the I amide bond (in the range of 1700 cm^−1^–1600 cm^−1^ [63]) was an indicator that the cross-linking mechanism of hyaluronic acid and ε-polylysine was ionic/electrostatic interaction. Furthermore, the ratio of NH_3_^+^/NH_2_ (the concentration of free NH_3_^+^ groups in ε-PL side chains [41]) was calculated from normalized FT-IR spectrum of all HA/ε-PL ratios. By increasing the ε-PL content in the hydrogel samples, the ratio of NH_3_^+^/NH_2_ increased from 0.66 in case of HA/ε-PL 90:10 up to 0.85 in case of HA/ε-PL 30:70. As it was foreseen that the antibacterial properties will be improved by increasing the ratio of NH_3_^+^/NH_2_ [41], the results were in good agreement with antimicrobial test results that revealed the increase in antibacterial efficiency with an increase of ε-PL content in samples (see Section 3.6).

### 3.5. Influence of HA/ε-PL Ratio on the Hydrogel Morphology

**Figure 5:**
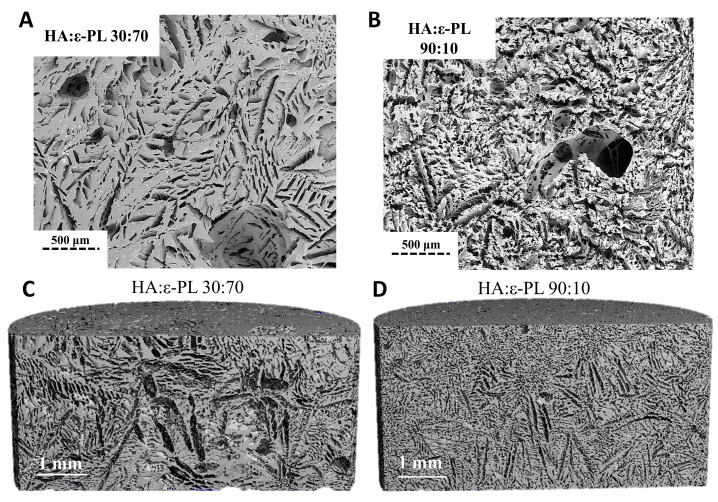
(**A**,**B**) SEM microphotographs of HA/ε-PL composites (**C**,**D**) μCT microphotographs of HA/ε-PL composites.

SEM was used to visualise the surface and cross-section morphology of lyophilized HA/ε-PL hydrogels (30:70 and 90:10 HA/ε-PL). HA/ε-PL composite cross-section analysis (Figure 5A,B) revealed the interconnected porosity, with average pore size ranging from 85 µm up to 267 µm. The amount of hyaluronic acid in the prepared HA/ε-PL composite did not affect the cross-sectional microstructure. Considering that the pore size ranging from 70 μm up to 500 μm is found to be optimal for the tissue engineering [64], the prepared hydrogels can be used for cartilage, as well as for wound treatment. The surface and cross-section morphology of lyophilized hydrogel samples were also observed by μ-CT (Figure 5C,D). Furthermore, μ-CT was used to determine the porosity of physically crosslinked hydrogels. Obtained results showed that with the change of HA/ε-PL ratio, the porosity of hydrogels has changed, but no coherent effect was observed (Table 1). By observing the cross-section of the samples, it was found that all HA/ε-PL hydrogel series have interconnected pores. The presence of interconnected pores is considered to be highly beneficial for nutritional supply in deeper scaffold areas, which are enabling cells to survive.

### 3.6. Antimicrobial Properties of HA/ε-PL Hydrogels

Antimicrobial properties of ɛ-PL against Gram-positive, Gram-negative bacteria and fungi have been previously proven [28,29]. Shortly, antimicrobial properties have been directly connected with the concentration of free amino (-NH_3_^+^) groups in the peptide structure. The positively charged NH_3_^+^ groups electrostatically interact with the negatively charged cell membrane [27,28], consequently causing damage to the cell membrane [29], which results in positive antimicrobial activity. Hence, the higher the concentration of protonated NH_3_^+^ groups in the polymer structure, the higher the antimicrobial activity.

**Figure 6:**
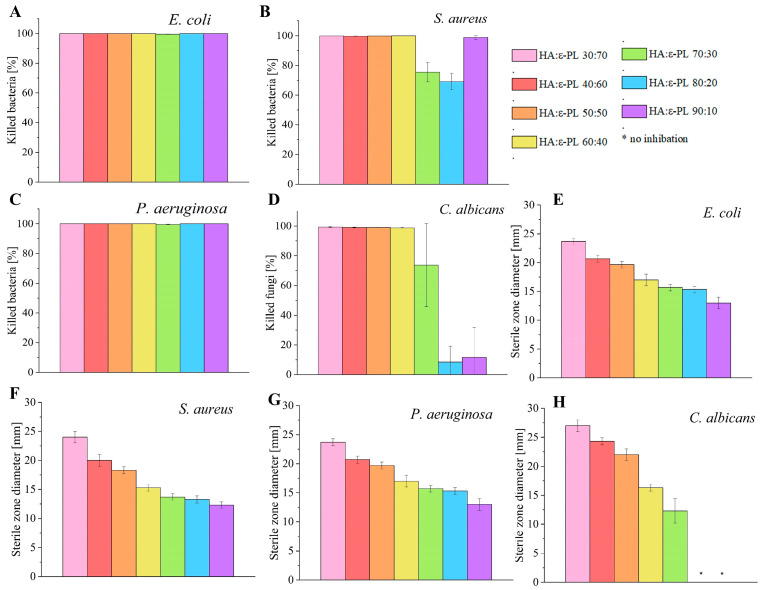
(**A**–**D**) HA/ε-PL hydrogels killing efficiency after one hour contact time with different microorganisms (**E**–**H**) Sterile zone diameter (mm) against various microorganisms.

All tested HA/ɛ-PL hydrogels demonstrated antimicrobial activity against Gram-positive (*S. aureus*) and Gram-negative (*E. coli*, *P. aeruginosa*) bacteria. The diameter of inhibition zones (Figure 6E–H and Supplementary Material Figure S2) of all of the hydrogels ranged from 12.3 ± 0.6 mm to 24.0 ± 1.0 mm for bacteria. The diameter of inhibition zones with fungi *C. albicans* ranged from 12.3 ± 2.1 mm to 27.0 ± 1.0 mm for hydrogels in which HA content was from 30 wt% up to 70 wt% but samples with HA:ε-PL ratio of 80:20 and 90:10 did not show any zone of inhibition. During the experiment, it was found that by increasing the ε-PL concentration in prepared hydrogel samples, the attained antimicrobial activity was also increased. The aforementioned results coincided well with data observed in FT-IR analyses (see Section 3.4), as well as with the expectations found in the literature where the increased concentration of ε-PL (increased count of free NH_3_^+^) provided better antimicrobial activity. Once all of the results from all studied microorganisms were summed up and compared, out of all tested samples, HA/ɛ-PL hydrogel with the ratio of 30:70 had the highest antimicrobial activity, while HA/ɛ-PL 90:10 had the lowest record.

In order to test the bactericidal and fungicidal effect, the dynamic contact test was performed (Figure 6A–D). After a contact time of 1 h, the killing efficiency of the most active sample, HA/ɛ-PL 30:70, reached 100.000% for Gram-negative bacteria *E. coli* and *P. aeruginosa*, while for *S. aureus* the achieved maximum was 99.964 ± 0.023%. The most negligible effect on all three bacteria used in the experiments was found for HA/ɛ-PL 70:30 (killing efficiency from 75.452% for *S. aureus* to 99.508% for *P. aeruginosa* and 99.632% for *E. coli*). When the killing efficiency of the hydrogels was tested on *C. albicans*, HA/ɛ-PL 30:70, reached the value of 99.341 ± 0.327%. Following these results, the effect of HA/ɛ-PL 70:30 was not significantly different from samples with higher ɛ-PL concentrations (*p* > 0.01) (killing efficiency of 73.721 ± 27.951%), possibly due to the data dispersion. In contrast, HA/ɛ-PL 80:20 and HA/ɛ-PL 90:10 demonstrated a fungicidal effect of only 8.462% and 11.615%, respectively, when used on *C. albicans*. The high antimicrobial activity of hydrogels has been ascribed to the similar composition of ɛ-PL. These results once again corroborated that the availability of charged amino (NH_3_^+^) groups of ε-PL is the main reason for the antimicrobial efficacy. However, one exception has been observed, HA/ɛ-PL 90:10 was more effective than HA/ɛ-PL 70:30 under dynamic test conditions for Gram-positive bacteria *S. aureus*. The main reason for this effect can be accredited to the sole structure of the hydrogel (i.e., its partial dissolution due to the high content of HA and immediate bioavailability of ɛ-PL).

### 3.7. In Vitro Cell Biocompatibility of HA/ε-PL Hydrogels

#### 3.7.1. Biocompatibility and Hemocompatibility of Hydrogels

**Figure 7:**
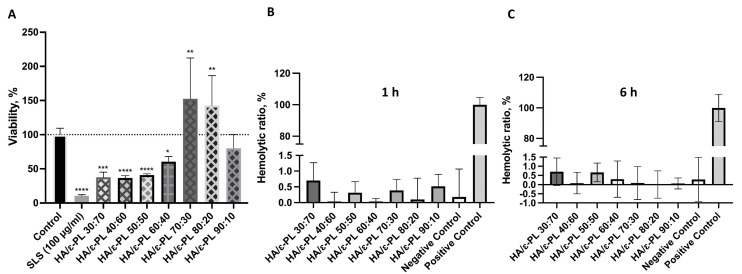
(**A**) Viability of Balb/c 3T3 cells after 24 h incubation with hydrogels in direct test. SLS—sodium lauryl sulphate (cytotoxicity control). Data graphs include some of our previously reported results (for HA/ε-PL 40:60, HA/ε-PL 50:50, HA/ε-PL 60:70 [41]); the dotted line represents the control level (100%); *n* = 5, except for HA/ε-PL 70:30, HA/ε-PL 80:20 and HA/ε-PL 90:10 in direct test (**A**) where *n*=6. * *p* < 0.05, ** *p* < 0.01, *** *p* < 0.001, **** *p* < 0.0001. Haemolytic activity of hydrogels expressed as a hemolytic ratio (%) after 1 h (**B**) and 6 h (**C**) incubation with diluted human blood samples. N = 3.

In order to consider any material for a potential medical application, in vitro cell biocompatibility should be evaluated. In the direct cytotoxicity assay, hydrogel samples were tested against subconfluent (60–70%) Balb/c 3T3 cell culture and incubated for 24 h. It was noticed that viability and confluence of the cells was less than 60% in the presence of hydrogel samples containing >40 wt% of ɛ-PL, if compared to the control that was sodium dodecyl sulphate as positive (cytotoxic) control and cells incubated without samples as negative (biocompatible) control (Figure 7A). For samples HA/ɛ-PL 30:70, HA/ɛ-PL 40:60 and HA/ɛ-PL 50:50 average cell viability was 37.64%, 36.67% and 40.75%, respectively. Whereas incubation with HA/ɛ-PL 60:40 sample resulted in 60.53% viability. The highest viability was observed for sample HA/ɛ-PL 70:30 (152.57%), followed by HA/ɛ-PL 80:20 (142.67%), while for the HA/ε-PL 90:10 viability was 79.98%. Therefore, following the cell viability trend it can be clearly seen that the increase of HA concentration (up until 70 wt%) in the prepared hydrogels led to higher cell viability. Differences in the results for high HA content samples (80 and 90 wt%) could be connected to the mechanical characteristics of the material—the higher the HA content, the less attractive the structure might be for cell growth. Similarly, the decrease of cell viability for samples containing ε-PL from 40 wt% up to 70 wt%, could be associated with polylysine bactericidal and fungicidal properties. Statistical significance between all ratios of prepared compositions was determined using ANOVA (*p* < 0.05), and the viability was statistically different, in comparison to the control, for all of the samples. Furthermore, it should be noted that samples HA/ε-PL 80:20 and HA/ε-PL 90:10 swelled considerably. In the case of HA/ε-PL 90:10 swelling in cultivation media might have also interfered with gas and nutrient exchange and thus resulted in slightly reduced cell viability. Furthermore, swelling of the HA also interfered with the extraction of neutral red accumulated in the viable cells, which led to higher variations in the results.

Additional to the cytotoxicity assessment with the direct contact test, hydrogel extracts were tested (Figure S3). Hydrogels containing up to 70% HA were extracted with the cell cultivation media at the ratio of 1:5 and were tested at concentrations of 12.5%, 25% and 50%. Hydrogels that contained 80 wt% and 90 wt% HA were extracted at ratios 1:20 and 1:50, respectively, due to the swelling, and the extracts were tested at 100%, 50% and 25% concentrations. Results were in line with those of the direct contact test—hydrogels with higher ɛ-PL content have a more negative effect on cell viability. Extracts of HA/ε-PL 30:70 and HA/ε-PL 40:60, in a concentration of 50% were the most toxic ones and they reduced the cell viability by 57.21% and 60.05%, respectively (Figure S3). When these extracts were further diluted, the cytotoxic effect decreased. In the case of HA/ε-PL 50:50 and HA/ε-PL 60:40, the highest concentration of extracts reduced viability by up to 40%. Differences between the three tested extract concentrations were less pronounced compared to the hydrogels with higher ɛ-PL content. The extract from the HA/ε-PL 70:30 did not reduce the cell viability by more than 20% in all of the tested concentrations. In the case of the extracts obtained from hydrogels that contained up to 70 wt% ε-PL, the changes in the cell viability were statistically significant compared to the control for all 50% and 25% extracts as well as for 12.5% HA/ε-PL 40:60 extract.

It was also found that undiluted HA/ε-PL 80:20 extracts reduced the cell viability by 32.78% (Figure S3). When diluted to 50% and 25%, viability was 75.43% and 90.16%, respectively. Reduction of the cell viability after the incubation with HA/ε-PL 80:20 100% and 50% extracts was statistically significant compared to the control. Extracts of HA/ε-PL 90:10 did not have any negative effects on cell viability.

Hemocompatibility is one of the main criteria for biomaterials that will be in direct contact with blood. During contact with the blood, the surface of the biomaterial should not cause adverse reactions, including coagulation, as well as activation or destruction of blood components such as platelets, leukocytes, erythrocytes etc. [65]. Obtained results showed (Figure 7B,C) that all tested hydrogels did not cause hemolysis of human blood. For all tested samples, hemolysis ratio was below 1.5%. Additionally, to evaluate if prolonged exposure could lead to hemolysis, 6 h incubation period was tested. However, no increase in hemolytic ratio was observed. Nevertheless, the differences among the samples were observed. These results indicated that prepared hydrogels did not have lytic effects on red blood cells and that the negative effects of some specimens (on the viability of actively dividing cells as fibroblasts) might be explained by the interference of the hydrogel components on cellular metabolism. However, further studies are needed to fully claim the connections with the polymer structures.

Overall, the obtained results indicated that with increasing ε-PL content safety and biocompatibility of hydrogels decreased. We assume that cytotoxic effects of hydrogel extracts with high ε-PL content could be attributed to the toxicity of high leaking concentration of freely positively charged NH_3_^+^, which induces cell membrane damage.

#### 3.7.2. Fluorescence Microscopy

**Figure 8:**
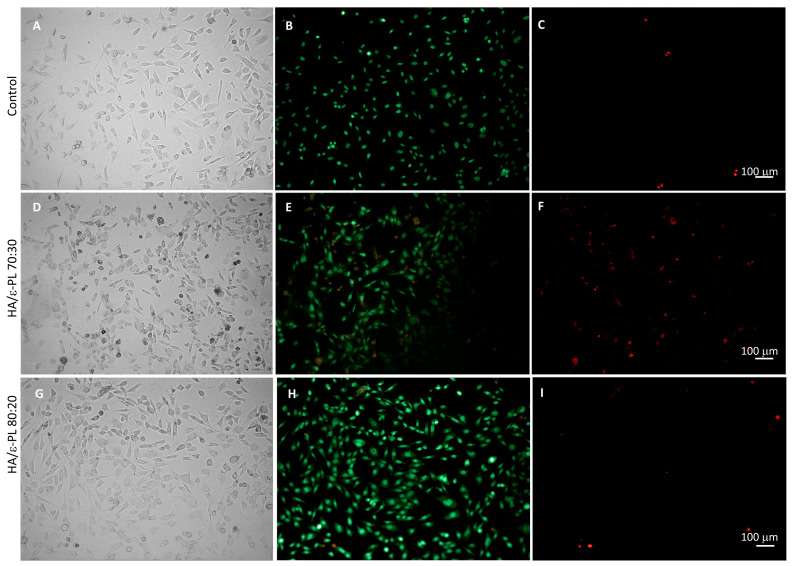
Live-dead staining of Balb/c 3T3 cells after 24 h incubation with hydrogel samples. (**A**–**C**): control, (**D**–**F**): HA/ε-PL 70:30, (**G**–**I**): HA/ε-PL 80:20; Brightfield (**A**,**D**,**G**), SYTO 9 (green) (**B**,**E**,**H**), propidium iodide (red) (**C**,**F**,**I**).

Microscopy of the cell cultures incubated with the neutral red (prior to the dye extraction) substantiate the above-described effects on their viability (Figure S4). High cell densities and accumulation of neutral red were observed for HA/ε-PL 90:10, HA/ε-PL 80:20 and HA/ε-PL 70:30, whereas incubation with HA/ε-PL 60:40 extracts resulted in lower densities of viable cells. In the presence of 50% extract of HA/ε-PL 70:30, cell viability was reduced more than in the case of 25% extract, but for the HA/ε-PL 80:20 sample in the presence of 100% extract, the density of the viable cells was high. However, these findings did not fully match the spectrophotometric readings of neutral red dye accumulation. This difference may be due to the interference from the leaked HA in the extraction media affecting the measurement of the dye. To further investigate the effects of hydrogels on the viability of mammalian cells, live-dead staining was performed. Hydrogel samples HA/ε-PL 70:30, HA/ε-PL 80:20 and HA/ε-PL 90:10 that produced the highest cell viability in the direct test were chosen for the live-dead staining. The results showed that cell confluences are similar to the control, with a slightly higher cell density in the presence of HA/ε-PL 80:20 (Figure 8) and HA/ε-PL 90:10 samples (Figure S5). The high density of the viable cells was observed after the incubation with all samples. However, a slightly higher number of dead cells was observed in the cell cultures incubated with HA/ε-PL 70:30 hydrogel (Figure 8). This contradicts the neutral red uptake measurements and could be explained by interference of the hydrogel with the neutral red dye extraction and absorption measurement that is most probably due to the swelling of the samples. These results emphasized the necessity for various complementing methodologies for a comprehensive characterization of hydrogels’ impact on the cell viability and proliferation.

## 4. Conclusions

The results of this study have demonstrated the potential of using hyaluronic acid (HA) and ε-polylysine (ε-PL) to develop physically crosslinked hydrogels that are free from toxic crosslinking agents or residues. The impact of HA content on the properties of the hydrogels was evaluated using various chemical, physical, and biological characterization methods. The results showed that the HA content significantly influenced the swelling behaviour, gel fraction, injection ability, mechanical stability, cell response as well as antimicrobial activity of the hydrogels. The minimum amount of HA needed to create a stable HA/ε-PL hydrogel was 40 wt%, while increased HA content resulted in elevated mechanical properties, decreased hydrogel stability in higher deformation values and improved cell biocompatibility. The FT-IR data confirmed the electrostatic/ionic interaction between HA and ε-PL as the crosslinking mechanism. All HA/ε-PL hydrogels showed self-healing properties and exhibited antimicrobial properties against Gram-positive and Gram-negative bacteria, as well as fungi *C. albicans*. As the concentration of ε-PL increased, the antimicrobial properties of the HA/ε-PL hydrogels improved but concurrently resulted in decreased cell viability. While hydrogels containing 50 wt% or less of HA were deemed to possess the greatest potential as injectable biomaterials, the optimal balance between antimicrobial efficiency and cell viability was found in the HA/ε-PL 70:30 and HA/ε-PL 80:20 compositions. Finally, the obtained results provided the new perception into development of new, patient-safe, and environmentally friendly biomaterials with great potential in minimally invasive surgical procedures able to reduce local infection risks.

## Figures and Tables

**Table 1:** The porosity of hydrogels depending on the HA/ε-PL ratio.

HA/ε-PL Ratio	Porosity ± STDEV, %
30:70	71.08 ± 0.65
40:60	69.38 ± 0.79
50:50	70.35 ± 0.18
60:40	73.53 ± 0.54
70:30	82.49 ± 0.58
80:20	76.69 ± 1.5
90:10	82.28 ± 0.48

## Data Availability

The data presented in this study are available on request from the corresponding author.

## Supplementary Materials

The following supporting information can be downloaded at: https://www.mdpi.com/article/10.3390/polym15081915/s1, Figure S1: Self-designed injectability equipment. Made for Tinius Olsen 25 ST (Horsham, PA), a multifunctional, mechanical materials testing machine; Figure S2: Representative sterile zones of HA/ε-PL hydrogels; Figure S3: Viability of Balb/c 3T3 cells after 24 h incubation with hydrogels extract test. Data graphs include some of our previously reported results (for HA/ε-PL 40:60, HA/ε-PL 50:50, HA/ε-PL 60:70 [41]); the dotted line represents the control level (100%); *n* = 5. * *p* < 0.05, ** *p* < 0.01, **** *p* < 0.0001. Figure S4: Representative images of Balb/c 3T3 cell cultures after incubation with hydrogel samples and staining with neutral red viability dye. 100× magnification. Figure S5: Live-dead staining of Balb/c 3T3 cells after 24 h incubation with hydrogel samples. (A–C): control, (J–L): HA/ε-PL 90:10. Brightfield (A,J), SYTO 9 (green) (B,K), propidium iodide (red) (C,L). The scale bar represents 100 µm.

## References

[1] Mathew A.P., Uthaman S., Cho K.H., Cho C.S., Park I.K. (2018). Injectable hydrogels for delivering biotherapeutic molecules. Int. J. Biol. Macromol..

[2] Liao Y., He Q., Zhou F., Zhang J., Liang R., Yao X., Bunpetch V., Li J., Zhang S., Ouyang H. (2020). Current Intelligent Injectable Hydrogels for In Situ Articular Cartilage Regeneration. Polym. Rev..

[3] Bueno E.M., Glowacki J. (2009). Cell-free and cell-based approaches for bone regeneration. Nat. Rev. Rheumatol..

[4] Sternberg K. (2009). Current requirements for polymeric biomaterials in otolaryngology. GMS Curr. Top. Otorhinolaryngol. Head Neck Surg..

[5] Collins M.N., Birkinshaw C. (2008). Physical properties of crosslinked hyaluronic acid hydrogels. J. Mater. Sci. Mater. Med..

[6] Jin Y., Koh R.H., Kim S.-H., Kim K.M., Park G.K., Hwang N.S. (2020). Injectable anti-inflammatory hyaluronic acid hydrogel for osteoarthritic cartilage repair. Mater. Sci. Eng. C.

[7] Wang L., Dong S., Liu Y., Ma Y., Zhang J., Yang Z., Jiang W., Yuan Y. (2020). Fabrication of Injectable, Porous Hyaluronic Acid Hydrogel Based on an In-Situ Bubble-Forming Hydrogel Entrapment Process. Polymers.

[8] Wang L., Li J., Zhang D., Ma S., Zhang J., Gao F., Guan F., Yao M. (2020). Dual-enzymatically crosslinked and injectable hyaluronic acid hydrogels for potential application in tissue engineering. RSC Adv..

[9] Lyu Y., Xie J., Liu Y., Xiao M., Li Y., Yang J., Yang J., Liu W. (2020). Injectable Hyaluronic Acid Hydrogel Loaded with Functionalized Human Mesenchymal Stem Cell Aggregates for Repairing Infarcted Myocardium. ACS Biomater. Sci. Eng..

[10] Ahsan A., Farooq M.A., Parveen A. (2020). Thermosensitive Chitosan-Based Injectable Hydrogel as an Efficient Anticancer Drug Carrier. ACS Omega.

[11] Alinejad Y., Adoungotchodo A., Grant M.P., Epure L.M., Antoniou J., Mwale F., Lerouge S. (2019). Injectable Chitosan Hydrogels with Enhanced Mechanical Properties for Nucleus Pulposus Regeneration. Tissue Eng. Part A.

[12] Tao J., Zhang Y., Shen A., Yang Y., Diao L., Wang L., Cai D., Hu Y. (2020). Injectable Chitosan-Based Thermosensitive Hydrogel/Nanoparticle-Loaded System for Local Delivery of Vancomycin in the Treatment of Osteomyelitis. Int. J. Nanomed..

[13] Ghosh M., Halperin-Sternfeld M., Grinberg I., Adler-Abramovich L. (2019). Injectable Alginate-Peptide Composite Hydrogel as a Scaffold for Bone Tissue Regeneration. Nanomaterials.

[14] Zhou J., Zhang K., Ma S., Liu T., Yao M., Li J., Wang X., Guan F. (2019). Preparing an injectable hydrogel with sodium alginate and Type I collagen to create better MSCs growth microenvironment. e-Polymers.

[15] Yang X., Wang B., Sha D., Liu Y., Xu J., Shi K., Yu C., Ji X. (2021). Injectable and antibacterial ε-poly(l-lysine)-modified poly(vinyl alcohol)/chitosan/AgNPs hydrogels as wound healing dressings. Polymer.

[16] Sun A., He X., Li L., Li T., Liu Q., Zhou X., Ji X., Li W., Qian Z. (2020). An injectable photopolymerized hydrogel with antimicrobial and biocompatible properties for infected skin regeneration. NPG Asia Mater..

[17] Gong C., Lu C., Li B., Shan M., Wu G. (2017). Injectable dopamine-modified poly(α,β-aspartic acid) nanocomposite hydrogel as bioadhesive drug delivery system. J. Biomed. Mater. Res. Part A.

[18] Wang L., Chen Z., Yan Y., He C., Li X. (2021). Fabrication of injectable hydrogels from silk fibroin and angiogenic peptides for vascular growth and tissue regeneration. Chem. Eng. J..

[19] Yuan T., Li Z., Zhang Y., Shen K., Zhang X., Xie R., Liu F., Fan W. (2021). Injectable Ultrasonication-Induced Silk Fibroin Hydrogel for Cartilage Repair and Regeneration. Tissue Eng. Part A.

[20] Liu S., Liu X., Ren Y., Wang P., Pu Y., Yang R., Wang X., Tan X., Ye Z., Maurizot V. (2020). Mussel-Inspired Dual-Cross-linking Hyaluronic Acid/ϵ-Polylysine Hydrogel with Self-Healing and Antibacterial Properties for Wound Healing. ACS Appl. Mater. Interfaces.

[21] Han C., Zhang H., Wu Y., He X., Chen X. (2020). Dual-crosslinked hyaluronan hydrogels with rapid gelation and high injectability for stem cell protection. Sci. Rep..

[22] Li S., Pei M., Wan T., Yang H., Gu S., Tao Y., Liu X., Zhou Y., Xu W., Xiao P. (2020). Self-healing hyaluronic acid hydrogels based on dynamic Schiff base linkages as biomaterials. Carbohydr. Polym..

[23] Ščeglovs A., Salma-Ancane K. (2020). Novel Hydrogels and Composite Hydrogels Based on ԑ-Polylysine, Hyaluronic Acid and Hydroxyapatite. Key Eng. Mater..

[24] Zerbinati N., Sommatis S., Maccario C., Capillo M.C., Grimaldi G., Alonci G., Rauso R., Guida S., Mocchi R. (2021). Comparative Physicochemical Analysis among 1,4-Butanediol Diglycidyl Ether Cross-Linked Hyaluronic Acid Dermal Fillers. Gels.

[25] Lee J.H. (2018). Injectable hydrogels delivering therapeutic agents for disease treatment and tissue engineering. Biomater. Res..

[26] Rodrigues B., Morais T.P., Zaini P.A., Campos C.S., Almeida-Souza H.O., Dandekar A.M., Nascimento R., Goulart L.R. (2020). Antimicrobial activity of Epsilon-Poly-l-lysine against phytopathogenic bacteria. Sci. Rep..

[27] Shukla S.C., Singh A., Pandey A.K., Mishra A. (2012). Review on production and medical applications of ɛ-polylysine. Biochem. Eng. J..

[28] Yoshida T., Nagasawa T. (2003). Epsilon-Poly-l-lysine: Microbial production, biodegradation and application potential. Appl. Microbiol. Biotechnol..

[29] Tan Z., Shi Y., Xing B., Hou Y., Cui J., Jia S. (2019). The antimicrobial effects and mechanism of ε-poly-lysine against Staphylococcus aureus. Bioresour. Bioprocess..

[30] Alkekhia D., Shukla A. (2019). Influence of poly-l-lysine molecular weight on antibacterial efficacy in polymer multilayer films. J. Biomed. Mater. Res. Part A.

[31] Chen S., Huang S., Li Y., Zhou C. (2021). Recent Advances in Epsilon-Poly-L-Lysine and L-Lysine-Based Dendrimer Synthesis, Modification, and Biomedical Applications. Front. Chem..

[32] Li S., Chen N., Li Y., Li X., Zhan Q., Ban J., Zhao J., Hou X., Yuan X. (2020). Metal-crosslinked ɛ-poly-L-lysine tissue adhesives with high adhesive performance: Inspiration from mussel adhesive environment. Int. J. Biol. Macromol..

[33] Yang Q., Xie Z., Hu J., Liu Y. (2021). Hyaluronic acid nanofiber mats loaded with antimicrobial peptide towards wound dressing applications. Mater. Sci. Eng. C.

[34] Mayandi V., Wen Choong A.C., Dhand C., Lim F.P., Aung T.T., Sriram H., Dwivedi N., Periayah M.H., Sridhar S., Fazil M.H. (2020). Multifunctional Antimicrobial Nanofiber Dressings Containing ε-Polylysine for the Eradication of Bacterial Bioburden and Promotion of Wound Healing in Critically Colonized Wounds. ACS Appl. Mater. Interfaces.

[35] Fallacara A., Baldini E., Manfredini S., Vertuani S. (2018). Hyaluronic Acid in the Third Millennium. Polymers.

[36] Tracuma E., Loca D. (2020). Hyaluronic Acid/Polylysine Composites for Local Drug Delivery: A Review. Key Eng. Mater..

[37] Tian W.M., Hou S.P., Ma J., Zhang C.L., Xu Q.Y., Lee I.S., Li H.D., Spector M., Cui F.Z. (2005). Hyaluronic Acid–Poly-D-Lysine-Based Three-Dimensional Hydrogel for Traumatic Brain Injury. Tissue Eng..

[38] Amato G., Grimaudo M.A., Alvarez-Lorenzo C., Concheiro A., Carbone C., Bonaccorso A., Puglisi G., Musumeci T. (2020). Hyaluronan/Poly-L-lysine/Berberine Nanogels for Impaired Wound Healing. Pharmaceutics.

[39] Guo J., Wei C., Wang X., Hou Y., Guo W. (2021). An in situ mechanical adjustable double crosslinking hyaluronic acid/poly-lysine hydrogel matrix: Fabrication, characterization and cell morphology. Int. J. Biol. Macromol..

[40] Simonson A.W., Lawanprasert A., Goralski T.D.P., Keiler K.C., Medina S.H. (2019). Bioresponsive peptide-polysaccharide nanogels—A versatile delivery system to augment the utility of bioactive cargo. Nanomedicine Nanotechnology. Biol. Med..

[41] Salma-Ancane K., Sceglovs A., Tracuma E., Wychowaniec J.K., Aunina K., Ramata-Stunda A., Nikolajeva V., Loca D. (2022). Effect of crosslinking strategy on the biological, antibacterial and physicochemical performance of hyaluronic acid and ɛ-polylysine based hydrogels. Int. J. Biol. Macromol..

[42] Parhi R. (2017). Cross-Linked Hydrogel for Pharmaceutical Applications: A Review. Adv. Pharm. Bull..

[43] Zuidema J.M., Rivet C.J., Gilbert R.J., Morrison F.A. (2014). A protocol for rheological characterization of hydrogels for tissue engineering strategies. J. Biomed. Mater. Res. Part B Appl. Biomater..

[44] Vadala G., Russo F., Musumeci M., D’Este M., Cattani C., Catanzaro G., Tirindelli M.C., Lazzari L., Alini M., Giordano R. (2017). Clinically relevant hydrogel-based on hyaluronic acid and platelet rich plasma as a carrier for mesenchymal stem cells: Rheological and biological characterization. J. Orthop. Res..

[45] Xue Y., Chen H., Xu C., Yu D., Xu H., Hu Y. (2020). Synthesis of hyaluronic acid hydrogels by crosslinking the mixture of high-molecular-weight hyaluronic acid and low-molecular-weight hyaluronic acid with 1,4-butanediol diglycidyl ether. RSC Adv..

[46] Capanema N.S.V., Mansur A.A.P., de Jesus A.C., Carvalho S.M., de Oliveira L.C., Mansur H.S. (2018). Superabsorbent crosslinked carboxymethyl cellulose-PEG hydrogels for potential wound dressing applications. Int. J. Biol. Macromol..

[47] Op’t Veld R.C., Walboomers X.F., Jansen J.A., Wagener F.A.D.T.G. (2020). Design Considerations for Hydrogel Wound Dressings: Strategic and Molecular Advances. Tissue Eng. Part B Rev..

[48] Ilyin S.O., Kulichikhin V.G., Malkin A.Y. (2016). The rheological characterisation of typical injection implants based on hyaluronic acid for contour correction. Rheol. Acta.

[49] Hájovská P., Chytil M., Kalina M. (2020). Rheological study of albumin and hyaluronan-albumin hydrogels: Effect of concentration, ionic strength, pH and molecular weight. Int. J. Biol. Macromol..

[50] Zhang Y., Hu C., Xiang X., Diao Y., Li B., Shi L., Ran R. (2017). Self-healable, tough and highly stretchable hydrophobic association/ionic dual physically cross-linked hydrogels. RSC Adv..

[51] Devi VK A., Shyam R., Palaniappan A., Jaiswal A.K., Oh T.-H., Nathanael A.J. (2021). Self-Healing Hydrogels: Preparation, Mechanism and Advancement in Biomedical Applications. Polymers.

[52] Zerbinati N., Capillo M.C., Sommatis S., Maccario C., Alonci G., Rauso R., Galadari H., Guida S., Mocchi R. (2021). Rheological Investigation as Tool to Assess Physicochemical Stability of a Hyaluronic Acid Dermal Filler Cross-Linked with Polyethylene Glycol Diglycidyl Ether and Containing Calcium Hydroxyapatite, Glycine and L-Proline. Gels.

[53] Stojkov G., Niyazov Z., Picchioni F., Bose R.K. (2021). Relationship between Structure and Rheology of Hydrogels for Various Applications. Gels.

[54] Guimarães C.F., Gasperini L., Marques A.P., Reis R.L. (2020). The stiffness of living tissues and its implications for tissue engineering. Nat. Rev. Mater..

[55] Vo A., Doumit M., Rockwell G. (2016). The Biomechanics and Optimization of the Needle-Syringe System for Injecting Triamcinolone Acetonide into Keloids. J. Med. Eng..

[56] Rył A., Owczarz P. (2020). Injectability of Thermosensitive, Low-Concentrated Chitosan Colloids as Flow Phenomenon through the Capillary under High Shear Rate Conditions. Polymers.

[57] Cilurzo F., Selmin F., Minghetti P., Adami M., Bertoni E., Lauria S., Montanari L. (2011). Injectability Evaluation: An Open Issue. AAPS PharmSciTech.

[58] Lv J., Meng Y., Shi Y., Li Y., Chen J., Sheng F. (2020). Properties of epsilon-polylysine·HCl/high-methoxyl pectin polyelectrolyte complexes and their commercial application. J. Food Process. Preserv..

[59] Lopes T.D., Riegel-Vidotti I.C., Grein A., Tischer C.A., de Faria-Tischer P.C.S. (2014). Bacterial cellulose and hyaluronic acid hybrid membranes: Production and characterization. Int. J. Biol. Macromol..

[60] Sokolova M., Locs J., Loca D. (2016). Hyaluronan Hydrogel/Calcium Phosphates Composites for Medical Application. Key Eng. Mater..

[61] Carneiro J., Döll-Boscardin P.M., Fiorin B.C., Nadal J.M., Farago P.V., de Paula J.P. (2016). Development and characterization of hyaluronic acid-lysine nanoparticles with potential as innovative dermal filling. Braz. J. Pharm. Sci..

[62] Rozenberg M., Shoham G. (2007). FTIR spectra of solid poly-l-lysine in the stretching NH mode range. Biophys. Chem..

[63] de Campos Vidal B., Mello M.L.S. (2011). Collagen type I amide I band infrared spectroscopy. Micron.

[64] Jonnalagadda J.B., Rivero I.V., Dertien J.S. (2015). In vitro chondrocyte behavior on porous biodegradable poly (e-caprolactone)/polyglycolic acid scaffolds for articular chondrocyte adhesion and proliferation. J. Biomater. Sci. Polym. Ed..

[65] Weber M., Steinle H., Golombek S., Hann L., Schlensak C., Wendel H.P., Avci-Adali M. (2018). Blood-Contacting Biomaterials: In Vitro Evaluation of the Hemocompatibility. Front. Bioeng. Biotechnol..

