# Supplementary material for: Exploring the Interplay of Antimicrobial Properties and Cellular Response in Physically Crosslinked Hyaluronic Acid/ε-Polylysine Hydrogels
Source: Polymers (Basel). 2023 Apr 17;15(8):1915. doi: 10.3390/polym15081915 (PMC10141856; doi:10.3390/polym15081915)
Supplement: Supplementary file 1 [file polymers-15-01915-s001.zip › polymers-2279367-supplementary.pdf]

Supporting Information

Exploring the interplay of antimicrobial properties and cellular response in physically crosslinked hyaluronic acid/ε-polylysine hydrogels.

K.Aunina[1,2], A.Ramata-Stunda[3], I.Kovrlija[1,2], E.Tracuma[1,2], R. Merijs-Meri[4], V.Nikolajeva[3], D.Loca[1,2*]

[1]Rudolfs Cimdins Riga Biomaterials Innovations and Development Centre of RTU, Institute of General Chemical Engineering, Faculty of Materials Science and Applied Chemistry, Riga Technical University, LV-1007 Riga, Latvia

[2]Baltic Biomaterials Centre of Excellence, Headquarters at Riga Technical University, LV-1007 Riga, Latvia

[3]Department of Microbiology and Biotechnology, Faculty of Biology, University of Latvia, LV-1050 Riga, Latvia

[4]Institute of Polymer Materials, Faculty of Materials Science and Applied Chemistry, Riga Technical University, LV-1048 Riga, Latvia

[*]corresponding author: dagnija.loca@rtu.lv; Tel.: +371-67089628; Fax: +371-67089619

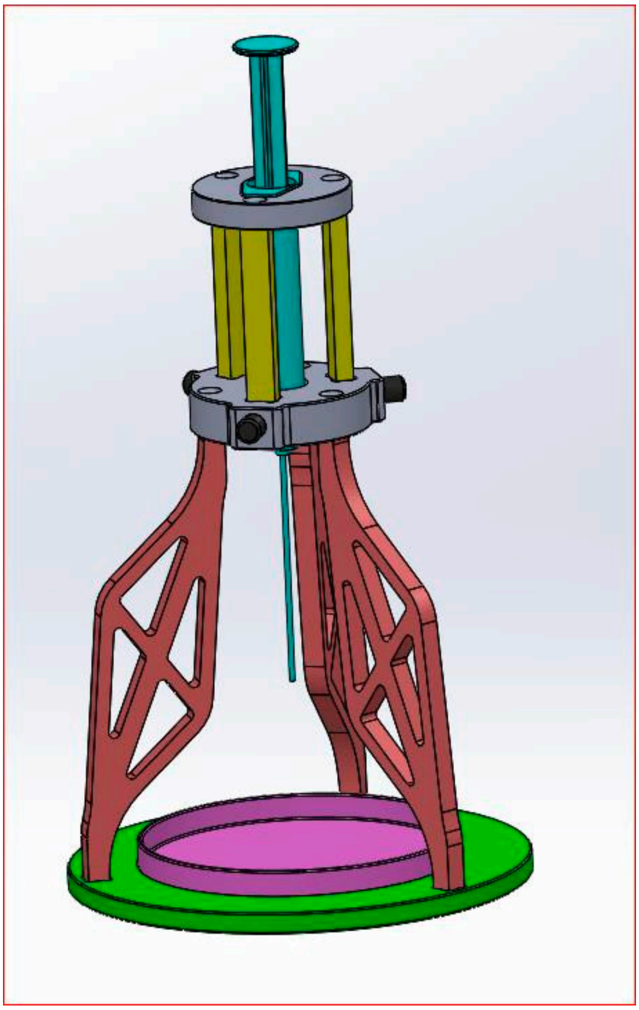

Figure S1. Self-designed injectability equipment. Made for Tinius Olsen 25 ST (Horsham, PA), a multifunctional, mechanical materials testing machine.

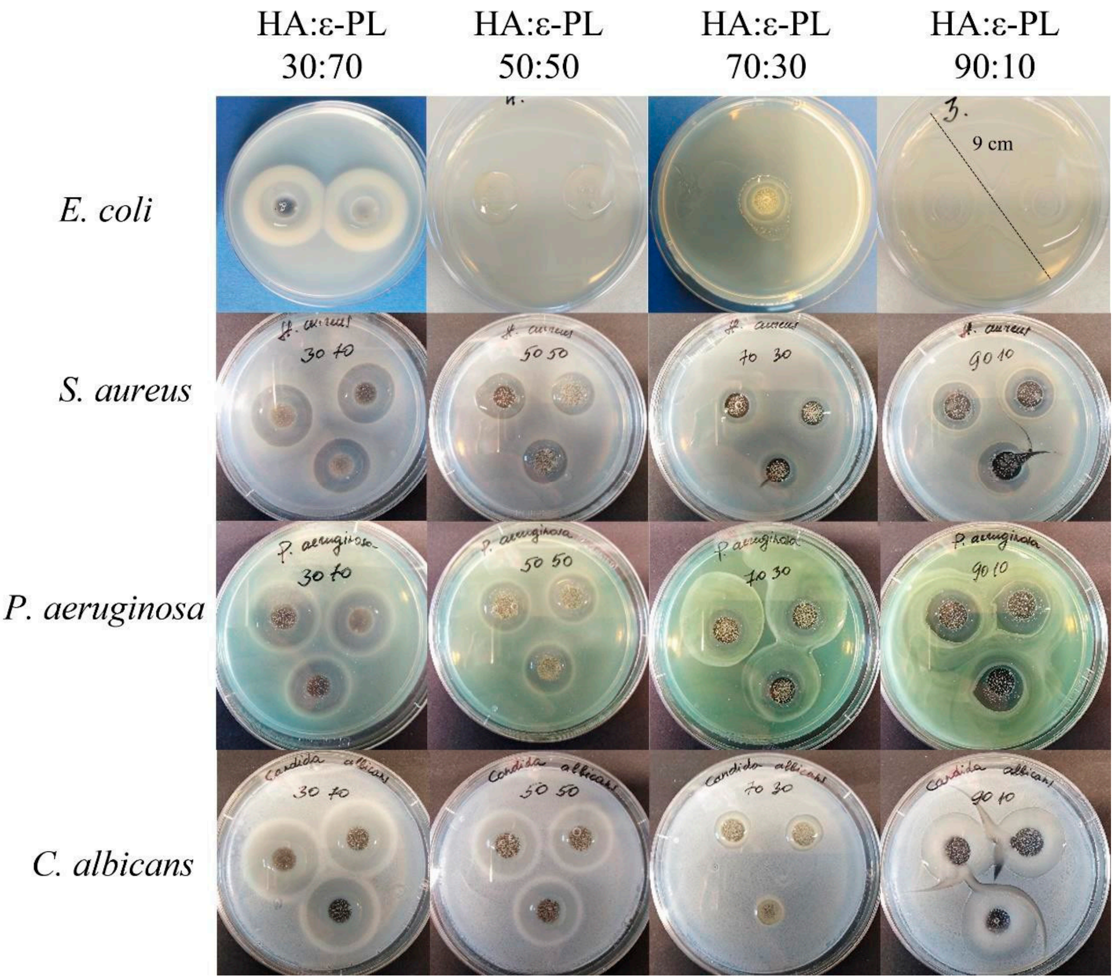

Figure S2. Representative sterile zones of HA/ε-PL hydrogels

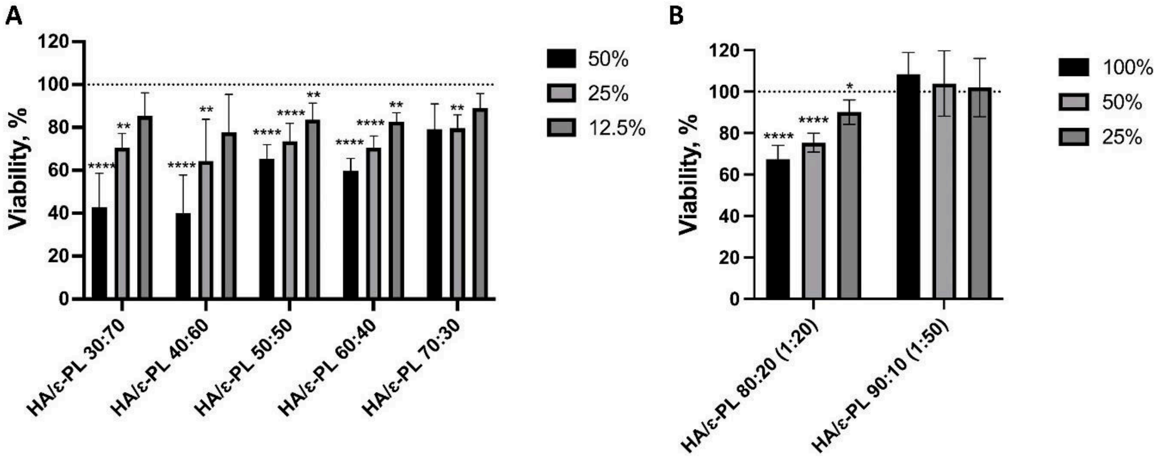

Figure S3. Viability of Balb/c 3T3 cells after 24 h incubation with hydrogels extract test. Data graphs include some of our previously reported results (for HA/ε-PL 40:60, HA/ε-PL 50:50, HA/ε-PL 60:70 [41]); the dotted line represents the control level (100%); n=5. * $p<0.05$, ** $p<0.01$, **** $p<0.0001$.

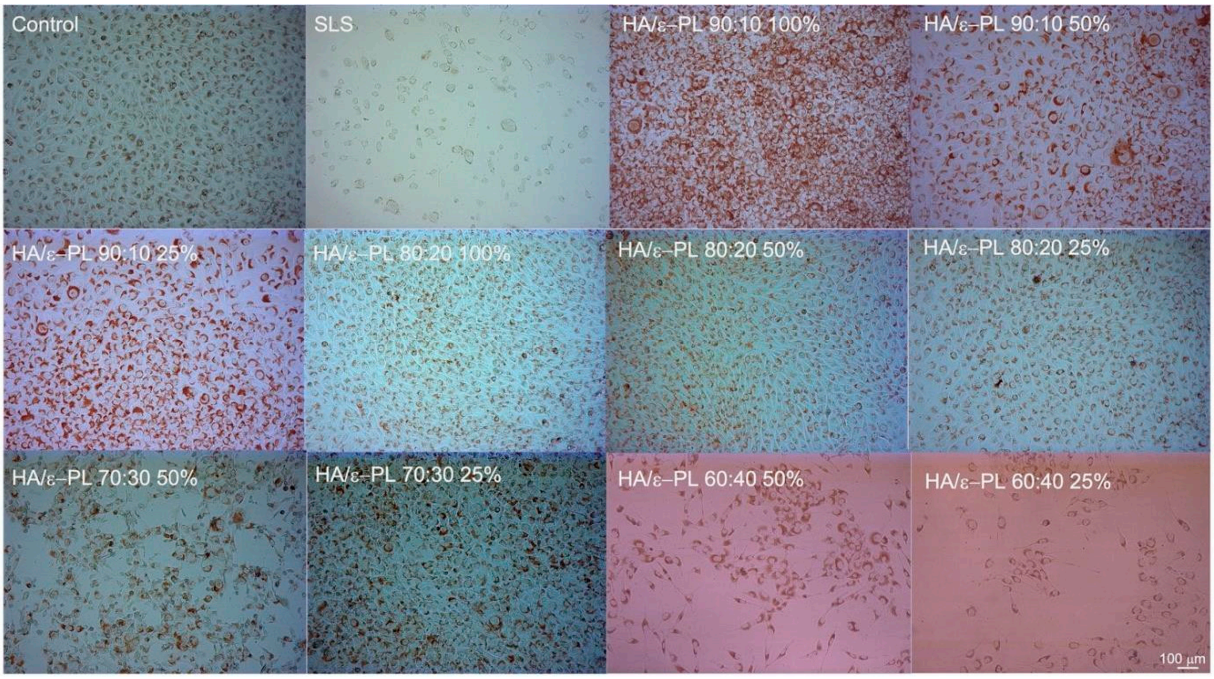

Figure S4. Representative images of Balb/c 3T3 cell cultures after incubation with hydrogel samples and staining with neutral red viability dye. 100x magnification.

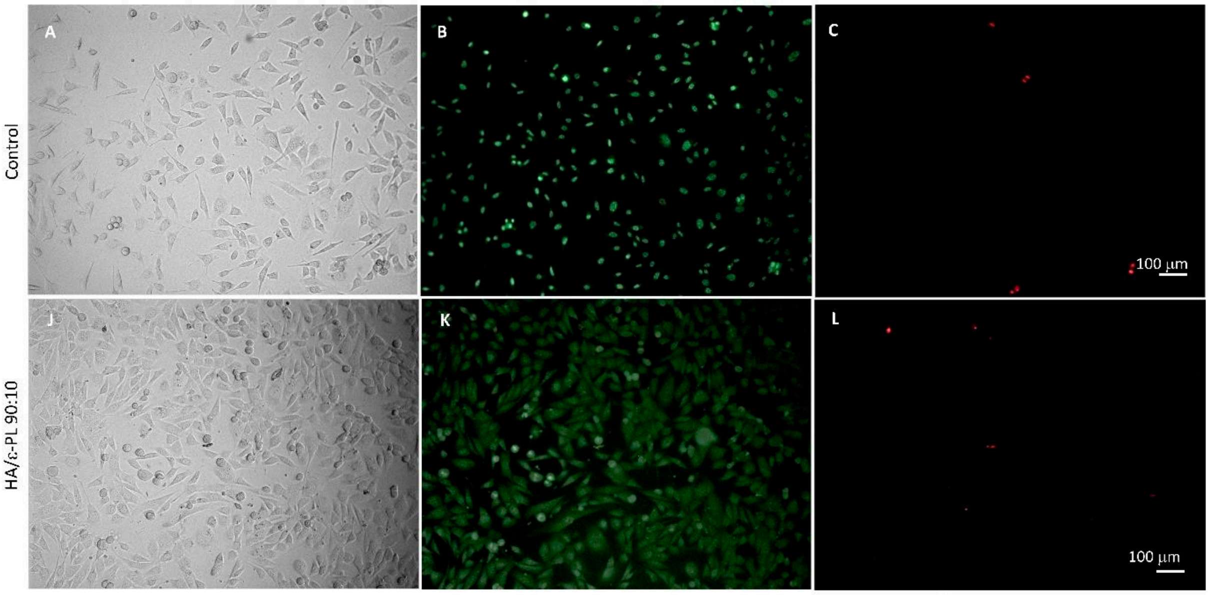

Figure S5. Live-dead staining of Balb/c 3T3 cells after 24 h incubation with hydrogel samples. A, B, C: control, J-L: HA/ε-PL 90:10. Brightfield (A, J), SYTO 9 (green) (B, K), propidium iodide (red) (C, L). The scale bar represents 100 μm.
